# Vitamin B12 and folate levels in healthy Swiss senior citizens: a prospective study evaluating reference intervals and decision limits

**DOI:** 10.1186/s12877-015-0060-x

**Published:** 2015-07-11

**Authors:** Martin Risch, Dominik W. Meier, Benjamin Sakem, Pedro Medina Escobar, Corina Risch, Urs Nydegger, Lorenz Risch

**Affiliations:** 1 Kantonsspital Graubünden, Zentrallabor, Loësstrasse 170, Chur, 7000 Switzerland; 2 Labormedizinisches Zentrum Dr. Risch, Waldeggstrasse 37, Liebefeld, 3097 Switzerland; 3 Private University Triesen, Triesen, 9495 Liechtenstein

**Keywords:** Age, Folate, Holotranscobalamin, Homocysteine, Glomerular filtration rate, Kidney function, Longitudinal study methylmalonic acid, Senescence, Reference interval, Vitamin B12

## Abstract

**Background:**

The vitamin B12 and folate status in nonanaemic healthy older persons needs attention the more so as decrease in levels may be anticipated from reduced haematinic provision and/or impaired intestinal uptake.

**Methods:**

A total of 1143 subjectively healthy Swiss midlands participants (637 females and 506 males), ≥60 years of age were included in this study. Levels of vitamin B12, holotranscobalamin (holoTC), methylmalonic acid (MMA), homocysteine (Hcy), serum folate, red blood cell (RBC) folate were measured. Further, Fedosov’s wellness score was determined. Associations of age, gender, and cystatin C/creatinine-based estimated kidney function, with the investigated parameters were assessed. Reference intervals were calculated. Further, ROC analysis was done to assess accuracy of the individual parameters in recognizing a deficient vitamin B12 status. Finally, decision limits for sensitive, specific and optimal recognition of vitamin B12 status with individual parameters were derived.

**Results:**

Three age groups: 60–69, 70–79 and ≥ 80 had median B12 (pmol/L) levels of 237, 228 and 231 respectively (p = 0.22), holoTC (pmol/L) of 52, 546 and 52 (p = 0.60) but Hcy (μmol/L) 12, 15 and 16 (p < 0.001), MMA (nmol/L) 207, 221 and 244 (p < 0.001). Hcy and MMA (both p < 0.001), but not holoTC (p = 0.12) and vitamin B12 (p = 0.44) were found to be affected by kidney function. In a linear regression model Fedosov’s wellness score was independently associated with kidney function (p < 0.001) but not with age. Total serum folate and red blood cell (RBC) folate drift apart with increasing age: whereas the former decreases (p = 0.01) RBC folate remains in the same bandwidth across all age groups (p = 0.12) A common reference interval combining age and gender strata can be obtained for vitamin B12 and holoTC, whereas a more differentiated approach seems warranted for serum folate and RBC folate.

**Conclusion:**

Whereas the vitamin B12 and holoTC levels remain steady after 60 years of age, we observed a significant increment in MMA levels accompanied by increments in Hcy; this is better explained by age-related reduced kidney function than by vitamin B12 insufficiency. Total serum folate levels but not RBC folate levels decreased with progressing age.

**Electronic supplementary material:**

The online version of this article (doi:10.1186/s12877-015-0060-x) contains supplementary material, which is available to authorized users.

## Background

The clinical significance of water soluble vitamin B12 (cobalamin) and of folate, and the importance of their routine analysis, is supported by recent findings in physiology regarding their function [1–8]. The simple yet biologically important role of support by these vitamins in catalysis of methyl group transfer is essential for life maintenance [9]. The function of folate overlaps with vitamin B12 both of them being essential in methylation reactions. With telomere shortening emerging as a research topic of senescence, it has been shown that the one-carbon metabolism pathway may affect telomere length through DNA-methylation [10].

Vitamin B12 deficiency later in life is estimated to affect 10 % of people over the age of 60 [11, 12]. Recent metaanalytic surveys [13, 14] confirmed the finding of vitamin B12-, and folate- deficiencies in elderly patients [15]. Optional but frequently practiced fortification of grain-based food supply is done with folic acid and vitamin B12 (www.blv.admin.ch).

Next to vitamin B12, folates are essential in eukaryotic cells for single carbon transfer reactions. Folate receptors transport their ligand via endocytosis [16] and jejunal folate resorption is of no clinical concern unless viral infection interferes [17]. In contrast the vitamin B12/gastric intrinsic factor cargo undergoes a risky trip down to ileal uptake involving vitamin B12 detachment from food proteins, and pancreatic protease scission of the vitamin B12-haptocorrin complex. There has recently been an upsurge of interest in the proper functioning of these interactions because of the extension of life expectancy and a concomitant increase in comorbidity, requiring updates in medical-geriatric check-ups [18]; early diagnosis in the elderly of vitamin deficiency is mandatory [19]. Some sources propose RBC folate a more stable indicator of folate sufficiency because of its intracellular confinement.

It is not enough to quantitatively estimate total vitamin B12 in order to test for vitamin B12 deficiency. It is true that MMA and Hcy are surrogate markers for vitamin B12 deficiency, and correctly indicate vitamin B12 failure to produce succinic acid and methionine. However, the effectiveness of these markers also depends on kidney function, which is now assessed by measuring the age-related impairment of glomerular filtration rate (eGFR: estimated equations) [20, 21]. In fact, kidney clearance of both MMA and Hcy is compromised in reduced kidney function [21].

Reference intervals (RI) for most laboratory analysis are now included by the providers of automatic instrument systems; their establishment is part of the brand and often calculated with serum banks of healthy blood donors in their work force age groups. In addition sex differences are issued only for relating analytes, mostly endocrinological.

It remains the responsibility of a laboratory to translate the RIs into good medical practice. RIs established with healthy sample donors from the region served by the medical laboratory need to be compared to those provided by the manufacturer of automated platforms and adjustments [22–24] made as required. The Swiss SENIORLAB study (www.seniorlabor.ch) is a study with over 1400 elderly participants allowing establishment of reference intervals of different analytes including vitamin D [25] and, analyzed here, vitamin B12/folate status.

## Methods

### Study population

Subjects were recruited from February 2009 to December 2011 in the context of the Swiss SENIORLAB study, which is an ongoing investigation in the canton of Berne (Switzerland) aimed at creating appropriate reference intervals (RI’s) of several analytes in the senior citizens (http://www.seniorlabor.ch). Subjectively healthy senior Caucasian volunteers aged 60 years and older were recruited. The study participants were contacted through newspaper advertisements, clubs and associations where there was a high probability that the membership would include healthy senior citizens (e.g., mountaineering clubs, sports clubs) and through personal contacts of those involved in organizing the study. A personal history of the patients was taken including specific information on medication and presence of several common diseases such as known cardiovascular disease, known diabetes mellitus, known thyroid disease, or known cognitive impairment. Anthropometric measurements were performed, and venous blood was drawn into S-Monovettes® (Sarstedt, Sevelen, Switzerland) after an overnight fasting period. In a longitudinal follow-up after baseline examination, all study participants were contacted and questioned on several issues regarding quality of life and survival. In the determination of reference intervals in elderly persons, survival can be employed to demonstrate a certain degree of health [26].

**Fig. 1:**
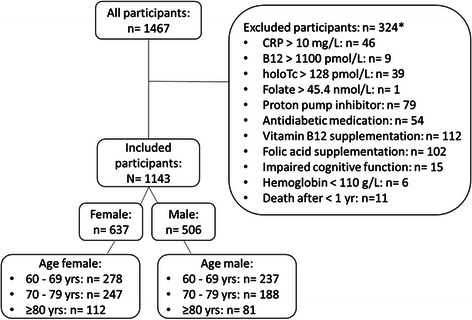
Chart representing all the recruited participants with those retained and those excluded from the study (n = number of participants). *Some participants fulfill several exclusion criteria

Of the 1467 participants, those with high C-reactive protein levels (>10 mg/L), vitamin B12-outliers (vitamin B12 > 1100 pmol/L), holoTC-outliers (holoTC >128 pmol/L), serum folic acid outliers (>45.3 nmol/L) or a hemoglobin suggesting anemia in the elderly (<110 g/L) [27] were excluded. Further exclusion criteria comprised the intake of proton pump inhibitors, antidiabetic medication, and vitamin B12 or folic acid supplementation as well as a clinical history suggesting the presence of cognitive impairment or a survival of less than one year (Fig. 1). Of the included study participants, 515 were between 60 and 69 years old, 435 between 70 and 79 years and 193 were 80 years old or older (Table 1). This study was in accordance with the Declaration of Helsinki and was approved by the cantonal institutional review board (KEK-Bern 166/08). All of the participants provided written informed consent.

**Table 1:** Baseline characteristics. The parameters given are associated with markers of vitamin B12 and folic acid status and are classified according to three different age groups. P-values are given for differences among the three different groups

	60-69 yrs	70-79 yrs	≥80 yrs	P values+
Female/Male (n)	278/237	247/188	112/81	0.53
Hb (g/L)	143.00 (136.00, 151.00); n = 515	142 (134, 149.00); n = 435	139 (131.00, 146.00); n = 193	<0.001
MCV (fl)	89.00 (87.00, 91.00); n = 515	90.00 (87.00, 92.00); n = 435	91.00 (88.00, 93.00); n = 193	<0.001
Fedosov’s ω	0.13 (−0.13, 0.36); n = 463	0.02 (−0.30, 0.33); n = 411	−0.10 (−0.39, 0.22); n = 184	<0.001
B12 (pmol/L)	237 (190, 292); n = 515	228 (180.00, 292); n = 435	231 (185, 301); n = 193	0.22
holoTc (pmol/L)	52.3 (41.0, 66.0); n = 508	54.1 (39.3, 69.0); n = 432	51.8 (38.2, 67.3); n = 193	0.60
MMA (nmol/L)	207 (169, 261.00); n = 467	221 (179, 294); n = 412	244 (190, 326); n = 184	<0.001
Hcy (μmol/L)	12.5 (10.5, 14.80); n = 515	13.9 (11.6, 16.7); n = 435	15.5 (12.8, 19.9); n = 193	<0.001
Serum folate (nmol/L)	19.5 (13.8, 27.0); n = 515	17.7 (12.9; 25.3); n = 4354	17.2 (13.0, 25.3); n = 193	0.01
Red cell folate (nmol/L)	899 (700,1157); n = 498	870 (669,1157); n = 431	837 (646, 1136); n = 192	0.17
Folate/B12	83.4 (58.9, 116.3); n = 515	81.5 (55.9, 112.0); n = 435	76.8 (57.3, 104.5); n = 139	0.09
eGFR (ml/min/1.73 m^2^)	90 (82, 98); n = 515	82 (69, 89); n = 435	67 (54, 78); n = 193	<0.001
Mentzer index	18.70 (17.60, 19.79); n = 515	18.94 (17.77, 20.22); n = 435	20.0 (18.33, 21.09); n = 193	<0.001

Footnote: Values are median and interquartile range (IQR) for continuous variables. n = number

^+^Kruskal-Wallis test for continuous variables; chi square test for proportion female/male

### Biochemical measurements

Analyses were performed on whole blood anticoagulated with EDTA, separated plasma therefrom or serum and kept at room temperature, on the same day of morning blood drawing. For CBC we use a Sysmex XE-5000 automated hematology analyzer (Sysmex, Horgen, Switzerland) providing 38 haematology values.

Total vitamin B12 and serum folic acid were assayed using the Abbott Architect i2000 analyzer (Abbott Diagnostics, Baar, Switzerland). Holotranscobalamin (holoTC) was assayed using an Axsym platform (Abbott Diagnostics, Baar, Switzerland). Because of restricted funds, only a partial sample (n = 1063) was analyzed on a SCIEX API 4000 LC-MS/MS system (AB Sciex, Brugg, Switzerland) for methyl malonic acid (MMA) [22, 28–32]. RBC folic acid was estimated using the relationship between red blood cell folate level, as measured with Beckman Unicel DxI 800 (Beckman Coulter, Nyon, Switzerland) and hematocrit. Creatinine was tested using an IDMS-standardized kinetic Jaffe method (Roche Diagnostics, Rotkreuz, Switzerland). The cystatin C levels and high sensitive CRP were determined using the nephelometric method with a Siemens Prospec instrument (Siemens Zurich, Switzerland). The α-amino acid homocysteine (Hcy) was measured using chemiluminescence on a Immulite 2000 instrument (Siemens, Zurich, Switzerland). The coefficient of variation in measuring the parameters of vitamin B12 and folic acid metabolism in our hands was 6.6 % for vitamin B12 (at a mean concentration of 162 pmol/L) 8.7 % for folic acid in serum (18.9 nmol/L), 6.8 % for holoTC (46 pmol/L), 9.3 % for Hcy (12.9 μmol/L), and for 1.8 % for MMA (390 nmol/L). Estimated glomerular filtration rate (eGFR) was computed using the combined CKD-EPI version involving creatinine and corrected cystatin C level (Chronic Kidney Disease Epidemiology Collaboration) [20, 33, 34]. The Mentzer index was calculated by dividing the RBC count into the MCV [35]. The Fedosov wellness quotient (ω; combined indicator of vitamin B12 status) was calculated according the formula: ω = log_10_(holoTC_n_) + log_10_(B12_n_) - log_10_(MMA_n_) - log_10_(Hcy_n_), where variables correspond to the combination of the variables into normalized concentrations; for example, holoTC_n_ = holoTC/holoTc_normal_ [36, 37]. The normal concentrations were obtained from reference [36]. Accuracy and precision of our assays comply with external and internal quality control requirements of the Swiss commission for quality assurance in the medical laboratory (QUALAB).

### Data analysis

We used descriptive statistical methods to define reference intervals and to cross-compare blood concentrations of different analyte values. To find a non-parametric association between selected analyte concentrations we sought for significance of Spearman rank order correlation. Reference intervals for total B12, holoTC, folate and RBC folate were calculated by means of the non-parametric method, as recommended by the CLSI guideline C28-A3c [38, 39]. The prevalence of vitamin B12 deficiency was assessed by Fedosov’s quotient [36]. The Kruskal-Wallis test was employed to evaluate differences in analyte concentrations among different age-groups (60–69, 70–79 and ≥80). Proportions were compared by chi-square test and chi-square test for trend. To characterize the diagnostic accuracy of the different analytes, receiver operating characteristic (ROC) curve analysis was performed; cutoff thresholds and ROC analysis of 4 separate markers of vitamin B12 deficiency (vitamin B12, holoTC, MMA, Hcy) was calculated assuming negative values ω < − 0,5 point to vitamin B12 deficiency [36]. Analogously to Fedosov [35], we used ROC curve analysis to determine the optimum decision threshold for the individual markers as lower cut-off for evaluation of a vitamin deficiency. We also determined the cut-offs, where an individual marker possesses a sensitivity or specificity of at least 99 %. As a marker of diagnostic accuracy, the areas under the curve of individual markers were compared according to DeLong et al. [40] Linear regression analysis was also done, in order to investigate a relationship between the metabolites Hcy and MMA with kidney function, independent of vitamin concentrations. The computer program Medcalc version 14.12.0 (Mariakerke, Belgium) was used for statistical calculations. Graphs were constructed by Medcalc and Graphpad Prism version 5.04 (GraphPad Software, La Jolla, USA).

## Results

### Vitamin B12 and surrogate markers

**Fig. 2:**
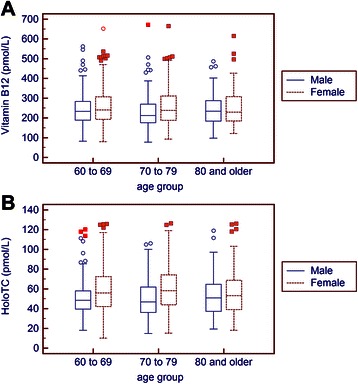
Vitamin B12 and holoTC serum levels in three arbitrarily defined advanced age groups classified according to sex. The box plot representation with vitamin B12 (panel **a**) and holoTC (panel **b**) as appearing in three age groups. The difference of vitamin B12 concentrations among males and females is significant in 70–79 year old (p = 0.001) and not significant in the participants aged 60–69 (p = 0.07) and ≥ 80 years (p = 0.69). The same comparisons of holoTC concentrations are significant for participants aged 60–69 and 70–79 (both p < 0.001), and not significant in the particpants ≥ 80 years (p = 0.77)

**Fig. 3:**
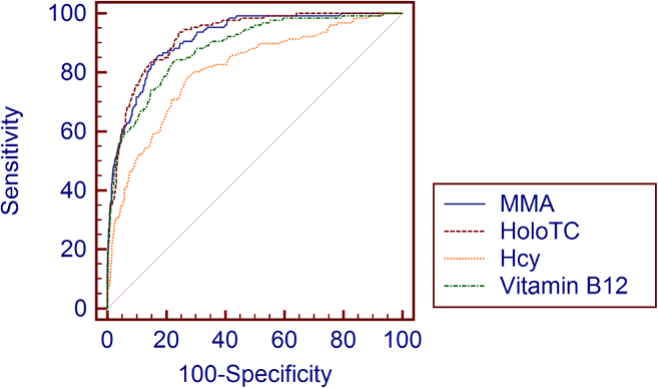
Receiver operating characteristic (ROC) curves of vitamin B12, holoTC, MMA and Hcy. The Fedosov wellness parameter ω = −0.5 was used as a separator of the groups assumed deficient and assumed healthy

Spearman rank correlations revealed statistically insignificant associations between vitamin B12 or holoTC and progressing years of age (p = 0.56 and p = 0.68). Total vitamin B12 serum levels and holoTC in the entire cohort thus remained stable over three arbitrarily defined age groups of 60–69, 70–79 and ≥80 year (Table 1). However, there were significant sex differences of holoTC in the age groups of 60–69 (p < 0.001), 70–79 years (p < 0.001), whereas no such differences could be detected in persons ≥ 80 years (p = 0.77) (Fig. 2 and Fig. 3). Therefore, a common reference interval for elderly persons could be determined for persons aged ≥80 year (21.3 (90 % confidence interval, CI, 19.3-24.0) – 118.2 (90 % CI 97–125.3) pmol/L), whereas sex-specific reference intervals can be given for persons from 60–79 years: 24.0 (90 % CI 21.0 – 25.6) – 115 (90 % CI 107.9 – 119.0) pmol/L in females and 22.3 (90 % CI and 21.0–24.5) – 94.4 (90 % CI 85.4–106.2) pmol/L for males. Because the 90 % CI’s overlap in both age groups and among both sexes, a combined lower limit of the reference interval might be justifiable and useful practical purposes. Because the upper limit of the reference interval is of minor clinical importance and the differences among age and sex groups can be regarded as small, the same practical considerations can be extraopolated to the common upper limit of the reference interval. A combined reference interval for both sexes and persons ≥ 60 years can thus be determined at 23 (90 % CI 21.9–24) – 109.3 (90 % CI 105 – 115) pmol/L.

When looking at sex differences of vitamin B12 concentrations in the 3 different age groups, significant differences between female and male participants can only be seen in the age group of 70–79 years (p < 0.001) (Fig. 2). Combined reference intervals for the age groups of 60–69 years and ≥ 80 year are: 124 (90 % CI 115–134) – 470 (90 % CI 428 – 507) pmol/L. The sex-specific reference intervals for the age group of 70–79 years are: 106 (90 % CI 77–122) – 443 (90 % CI 349–672) pmol/L for men and 113 (90 % CI 101–126) – 490 (90 % CI 442–511) pmol/L for females. As found above with holoTC the confidence intervals of the upper and lower limits overlap between the different groups. A single reference interval combining all age and sex groups for practical purposes thus also seems justifiable. A common reference interval for vitamin B12 in elderly persons thus can be determined at 119 (90 % CI 114–126) – 465 (90 % CI 442–497) pmol/L.

The prevalence of metabolic vitamin B12 deficiency [Fedosov’s wellness quotient ω < −0.5] in our cross-sectional survey, within the three age groups 60–69, 70–79 and ≥80 year were significantly different and showed a significant trend (p < 0.001 for both) as follows: 8.0 % (37/426), 13.4 % (55/356) and 19.0 % (35/149).

There was a statistically significant increase in serum concentrations of Hcy and MMA with advancing age (p < 0.001 for both analytes); because it is known that these analytes pass the glomerular barrier, their senescent increment depends on eGFR, which significantly deteriorated over the three age groups (p < 0.001) (Table 1). Additional file shows this in more detail [Additional file 1]. Using a linear regression model employing age, serum folate, vitamin B12, gender and eGFR as independent and Hcy or MMA as dependent variables revealed a highly significant (p < 0.001) inverse relationship of both Hcy and MMA with eGFR. It should be noted that both vitamin B12 (p = 0.44) and holoTC (p = 0.12) were unrelated to eGFR in univariate analysis but also in a regression model adjusting for age and gender [Additional file 2].

The Fedosov wellness quotient calculated from the four vitamin B12 dependent analytes exhibited a statistically weak inverse correlation with progressing age in univariate analysis (r = −0.153, p < 0.001) [41]. However, a linear regression model (R^2^ = 0.95) incorporating MMA, Hcy, holoTc, vitamin B12, eGFR and gender as independent and Fedosov’s wellness quotient as dependent variable reveals the four parameters of vitamin B12 status as well as kidney function to be independent predictors (p < 0.001 for all) of Fedosov’s wellness score. Age (p = 0.97) and gender (p = 0.59) did not have a significant association in this model.

To scrutinize the diagnostic accuracy of vitamin B12 dependent analyses, we performed a ROC analysis (Fig. 2). It appears that the largest area under the curve (AUC) is seen with holoTC: 0.923 (95 % CI 0.905-938), followed by MMA: 0.916 (95 % CI 0.897-0.932), followed by vitamin B12: 0.884 (95 % CI 0.863-0.903); Hcy reached an AUC value of 0.807 (95 % CI 0.782-0.831). A comparison of the AUC revealed holoTC to display significantly better diagnostic characterstics than vitamin B12 (p = 0.03) and homocysteine (p < 0.001). HoloTc (p < 0.001), MMA (p < 0.001) and vitamin B12 (p = 0.009) had significantly better diagnostic characteristics than Hcy. There were no differences in AUC between holoTC and MMA (p = 0.67) as well as between MMA and vitamin B12 (p = 0.14). As obtained by ROC curve analysis, the three decisive cut-offs (cut-off with 99 % specificity, cut-off with 99 % sensitivity, optimum decision threshold) for the different markers were: >25.8 pmol (specificity 99.04 %; sensitivity 32.81 %), ≤57 pmol/L (sensitivity 99.22 %; specificity 46.06 %), ≤43.2 pmol/L (sensitivity 93.75 %; specificity 75.85 %) for holoTC; >485 nmol/L (specificity 99.04 %; sensitivity 32.81 %), ≥216 nmol/L (sensitivity 99.22 %; specificity 55.85 %), ≥284 nmol/L (sensitivity 85.94 %; specificity 82.55 %) for MMA; ≤131 pmol (specificity 99.04 %; sensitivity 29.69 %), ≤316 pmol/L (sensitivity 99.22 %; specificity 20.21 %), ≤194 pmol/L (sensitivity 83.59 %; specificity 77.55 %) for vitamin B12; >9,5 μmol/L (specificity 11.17 %; sensitivity 99.22 %), ≥26.8 μmol/L (sensitivity 9.38 %; specificity 99.04 %), ≥15.1 μmol/L (sensitivity 77.34 %; specificity 72.87 %) for Hcy. As an alternative to reference intervals, a 2 cut-off model indicating probable vitamin B12 deficiency, probable sufficient vitamin B12 status as well as a greyzone can be derived from these cut-offs. For the 4 investigated parameters, these cut-offs are given in a separate table (Table 2).

**Table 2:** Cut-offs indicating probable vitamin B12 deficiency, probable vitamin B12 sufficiency and a grey zone of diagnostic uncertainty. These cut-offs are given for four markers of vitamin B12 status and were obtained by ROC-analysis

	Probable vitamin B12 deficiency	Grey zone	Probable vitamin B12 sufficiency
Vitamin B12 (pmol/L)	<131	131 – 315	≥316
Holotranscobalamin (pmol/L)	<25.8	25.8 – 56.9	≥57.0
MMA (nmol/L)	>485	217 – 485	≤216
Homocysteine (μmol/L)	>26.8	9.6 – 26.8	≤9.5

Together, the specific cut-offs of holoTC (25.8 pmol/L) and vitamin B12 (131 pmol/L) as determined by biochemical vitamin B12 status obtained by Fedosov’s wellness quotient and ROC curve analysis fall close to the 90 % CI of the lower limit of the combined reference interval for holoTC (21.9-24.0 pmol/L) and vitamin B12 (114-126pmol/L).

### Folate

The total serum folate decreased with increasing age (r = − 0.08; p = 0.01). Further, significant sex-specific differences can be seen in the age groups from 60–79 (p < 0.001) but not in individuals ≥80 years (p = 0.07). The sex-specific reference intervals in the age group 60–69 years are: 8.1 (90 % CI 6.3-8.8) – 40.8 (90 % CI 36.8-45.1) nmol/L for males and 9.6 (90 % CI 8.4-10.7) – 43.3 (90 % CI 42.3-44.6) nmol/L for females. In the age group 70–79 years, the sex-specific reference intervals are 6.7 (90 % CI 3.7-8.1) – 37.1 (90 % CI 34.5- 44.1) nmol/L in males and 9.1 (90 % CI 7.4-9.8) – 43.9 (90 % CI 40.4- 45.1) nmol/L in females. A combined reference interval for the age group ≥ 80 years can be determined at 8.0 (90 % CI 6.5 – 8.7) – 43.0 (90 % CI 39.2-45.1) nmol/L. A reference interval combining all gender and age strata for practical purposes reveals a lower limit of 8.2 (90 % CI 7.6-8.5) and an upper limit of 42.8 (90 % CI 41.4-43.8) nmol/L.

Interestingly, RBC folate remained stable over increasing age (r = − 0.045, p = 0.12), There was a significant difference in RBC folate levels in females and males (p = 0.02). Accordingly, gender specific reference intervals for all age groups were calculated. The reference interval was 452 (90 % CI 396–503) – 1885 (90 % CI 1735–2045) nmol/L for males and 435 (90 % CI 399–485) – 2067 (1903–2331) nmol/L for females. Finally, a reference interval combining all gender and age strata for practical purposes reveals a lower limit of 444 (90 % CI 408–480) and an upper limit of 1934 (90 % CI 1869–2066) nmol/L.

As age increased, the folate/vitamin B12 ratio tended to decrease (r = 0.06, p = 0.049), an observation in line with the observation that serum levels of folic acid recede with age by contrast with vitamin B12. Serum folate and Hcy were inversely correlated (r = − 0.485, <0.001). As a matter of fact, such correlation was better than the one resulting from comparison of vitamin B12 and Hcy (r_s_ = −0.253, p < 0.001). There was a significant correlation of serum folate with eGFR (r = 0.10; p < 0.001) [Additional file 2]. Such an association was absent with RBC folate (r = 0.01; p = 0.58) [Additional file 2]. In a linear regression model incorporating age, sex and eGFR as independent and serum folate as dependent variable maintained the independent relationship of eGFR and serum folate (p = 0.03).

## Discussion

Our study on subjectively healthy elderly Caucasian participants from a circumscribed geographical region shows for the first time that holoTC, but also vitamin B12 levels from 60 year onwards remain within RIs set by international expert panels with people from all ages [42, 43] whereas serum folate concentrations – though not RBC folate - recede to some extent. It could be shown that MMA, Hcy as well as Fedosov’s wellness quotient rather are associated with kidney function than with age. Finally, we were able to present reference intervals and decision limits for the different investigated markers in the elderly.

An overwhelming proportion of the literature implies that, with progressing age, vitamin B12 and folate levels in senior citizens fall short of currently validated RIs [44–48], a finding that is challenged by our data, obtained from participants apparently in good health and with a relatively uniracial background [49, 50]. Most other studies, on metaanalysis, reveal cohorts comprised of study subjects seeking treatment or already ill or even subject to multimorbidity [14, 47, 51, 52]. The recruitment of study subjects apparently devoid of cognitive impairment might further augment the proportion of those with maintained vitamin B12 and folate levels now known to impinge on cognitious performance ([44, 45, 53–55]. With the recent discoveries regarding the indispensable functions of vitamin B12 and folate in living organisms [1, 2, 7], it comes as little surprise that vitamin B12 and, to a lesser extent, folate, maintain their levels throughout our life cycle or else the participating subjects would not have been able to enter the study in advanced age under the classification ‘healthy’.

This was a community-dwelling cohort and haematinic sufficiency [56] can be assumed among participants whose BMI was normal and who were omnivorous, well fed and unlikely to be vegetarians. Marginal vitamin B12 haematinic provision remains a topic in daily health care. Switzerland counts only around 20.000 lactovegetarians distributed over all age groups: inadequate dietary intake of these two vitamins cannot be regarded as a relevant issue in this study. The effect of food fortifications on the levels of vitamin B12 and folate could not be addressed. With the Canadian Health Measures Survey on healthy folate replete subjects, the prevalence of folate deficiency was close to zero while vitamin B12 deficiency (cutoff < 148 pmol/L) came up to 5 % [43] but without effect of advanced age in older adults (60–79 years). In Switzerland, intentional fortification of food with vitamin B12 and folic acid is not enforced by law unless vitamin supplementation is a recommended component of pregnancy care. We thus might assume that dietary intake by the healthy elderly studied here is adequate.

The drop in folate but not RBC folate levels observed here may be linked to an intracellular maintenance of folic acid entrapped in RBCs but no full explanation can be forthcoming until half-life studies in both body compartments, plasma and RBC, have been conducted. Some authors hold that free and RBC folate are equivalent [57] and guidelines from the UK judge serum folate measurement as sufficient for clinical purpose [58]. Some are of the opinion that estimation of RBC folate is required to appreciate general tissue folate supply; should the latter view prevail for interpretation of our observations, then increasing age at least is not connected to folate deficiency.

In line with acknowledged understanding, our study links the surrogate markers of vitamin B12 status, i.e., MMA & Hcy, more closely to waning kidney function than to age. It is widely confirmed that with advancing age and particularly over age 50, functional kidney performance declines by > 1 ml/min/year/1.73 m^2^ eGFR [59–61]. Our cohort confirms receding kidney function with progressing age ≥60 years using state-of-the art evaluation with creatinine and corrected cystatin C levels [20]. Thus logic would dictate that there are limits to applicability of MMA and Hcy as surrogate markers in geriatrics. Unhelpfully, quite a few studies have used claims of high levels of MMA and Hcy to underscore their findings of vitamin B12/folate reductions without testing for impaired kidney function, which is now well acknowledged, to increase MMA and Hcy levels [62].

It is noteworthy that significantly lower, but still normal, vitamin B12 median levels emerge in elderly males by comparison with females in the 60–79 years age range. Although no such sex difference showed up in a Canadian-HMS cycle 1 [43], it was observed that elderly males clearly were at higher risk for Vitamin B12/folate deficiency in a NHANES survey on 1770 elderly persons [47] and in our study women maintain sufficient vitamin B12/folate levels from their reproductive life cycle onwards.

In our study we have seen an increment in MCV with increasing age. Although the index values steadily increase by merely 1 fl from the youngest to the oldest age group, the difference is statistically significant because of the narrow distribution. This observation confirms previous reports indicating a link between macrocytosis and poorer age-related cognitive performance [18]; the selection criteria prior to study entry used here preclude such an association in the cohort studied here [52]. Out of curiosity, we also estimated the Mentzer index, now undergoing a revival as a test for safety of stem cell apheresis from healthy donors [35]. The values here obtained confirm those observed with MCV and in our case served as a validity criterion for our observations.

In an earlier report on a small subsample of the present cohort, we reported a borderline difference of eGFR in participants with low and normal holoTC [38]. In line with other investigations, we now could demonstrate that holoTC is not influenced by kidney function [63]. The fact that we found holoTC to possess the highest accuracy in recognizing an insufficient vitamin B12 status together with the independence of the parameter from reduced kidney function (a problem often occurring in the elderly) and from age seems to make holoTC an attractive first line choice for assessing vitamin B12 status in the elderly.

There are several possibilities to interpret markers of vitamin B12 and folate status. The most often used approach is the comparison with reference intervals. We were able to report reference intervals for all relevant parameters relating to the investigation of vitamin B12 and folate status in the elderly. Each of the reported stratum provides results from far more than 120 subjects, a lower limit generally accepted for evaluation of valid reference intervals [39]. Reference intervals have been reported from substantially smaller collectives [22]. The present investigation to the best of our knowledge is the largest collective of elderly persons, where reference intervals for vitamin B12 and folate status have been derived in an integral approach.

Another way of interpreting laboratory results is the comparison with pathophysiological conditions. We also have taken this approach with ROC curve analysis, which allows to describe areas of relative certainty (i.e., above or below two cut-offs) together with a greyzone. Interestingly, the lower limits of the reference intervals are similar to the specific but insensitive cut-offs. Applying classically evaluated reference intervals for assessment of vitamin B12 deficiency can thus be regarded as very specific and relatively insensitive. Within the difficult context of diagnosing vitamin B12 and folate deficiency, we think that using an approach with decision limits provided in Table 2 is preferable to the use of reference intervals. Unfortunately, a functional score for detecting folate deficiency in analogy to the Fedosov wellness quotient in vitamin B12 deficiency is lacking so far.

Our study contains limitations:

(i) The medication and supplementation intake of the participants was self-reported. It might be that some patients had supplements and medications leading to erroneous inclusion of a participant in this analysis. We can therefore not rule out the possibility that a portion of our participants have normal vitamin B12 levels because supplemented. As impaired cognition was an exclusion criterion, it can be assumed that the number of affected individuals is low.
(ii) It is unfortunate that the questionnaire for study subjects did not include more details on eating habits

## Conclusions

Whereas the vitamin B12 and holoTC levels remain steady after 60 years of age, we observed a significant increment in MMA levels accompanied by increments in Hcy; the latter is better explained by age-related reduced kidney function than by vitamin B12 insufficiency. Total serum folate levels but not RBC folate levels decreased with progressing age. The present work evaluated decision limits for the use of these parameters in the elderly.

## Additional files

Additional file 1: **Estimated glomerular filtration rate of the individuals studied.** A statistically significant reduction (<0.001) in eGFR levels with increasing age was observed.
Additional file 2: **Association of folate, vitamin B12 and related markers with kidney function (eGFR).** The 6 panels show the serum concentrations of each of vitamin B12 (r=0.02;p=0.44), holoTC (r=−0.04; p=0.14) , MMA (r=−0.29; p<0.001), Hcy (r=−0.46; p<0.001), serum folate (r=0.10; p<0.001) and RBC folate (r=0.01; p=0.58) as compared to eGFR, plotted independently of age.

## Abbreviations

AUC: Area under the curve
CI: confidence interval
ECL: electronic chemiluminescence
GFR: glomerular filtration rate
Hcy: homocysteine
holoTC: holotranscobalamin
MCV: mean corpuscular volume
MMA: methylmalonic acid
OTC: over the counter (to denote: nonprescription drug)
PPI: proton pump inhibitor
RBC: Red Blood Cells
RI: Reference interval
ROC: Receiver operating characteristic
