# Supplementary figures and images for: Vitamin B12 and folate levels in healthy Swiss senior citizens: a prospective study evaluating reference intervals and decision limits
Source: BMC Geriatr. 2015 Jul 11;15:82. doi: 10.1186/s12877-015-0060-x (PMC4499201; doi:10.1186/s12877-015-0060-x)

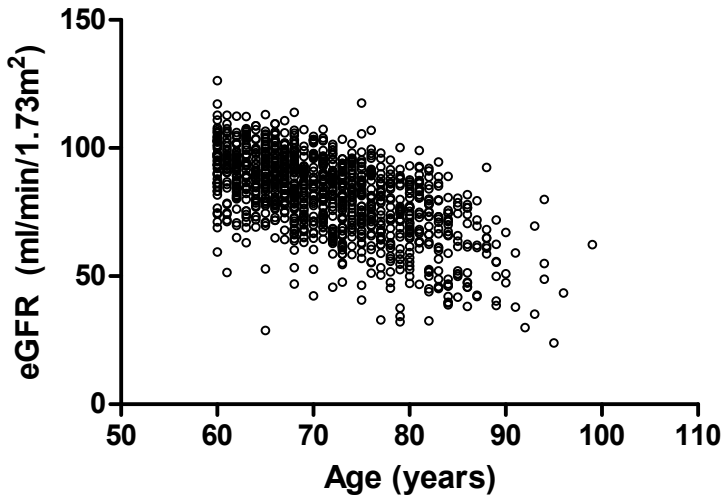

Supplement: Additional file 1: — Estimated glomerular filtration rate of the individuals studied. A statistically significant reduction (<0.001) in eGFR levels with increasing age was observed. [file 12877_2015_60_MOESM1_ESM.pdf]

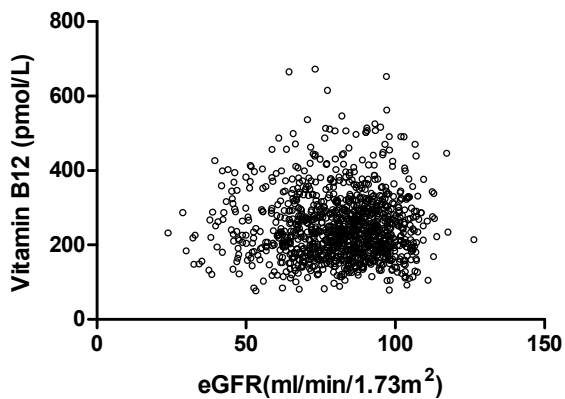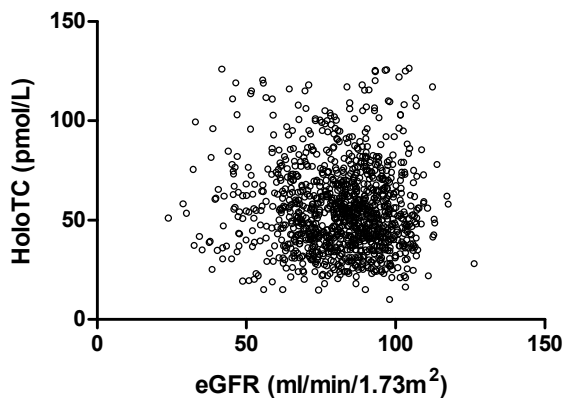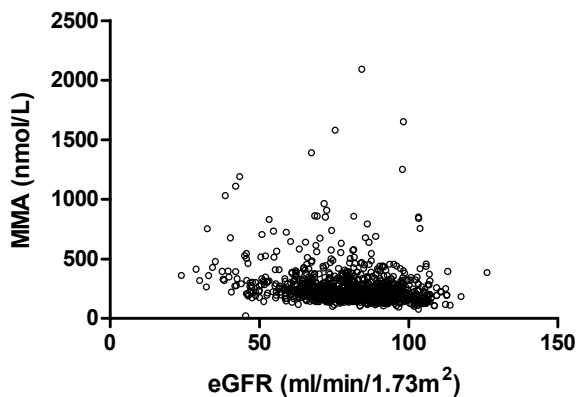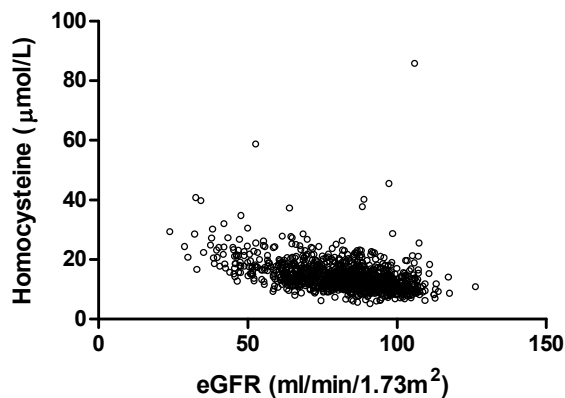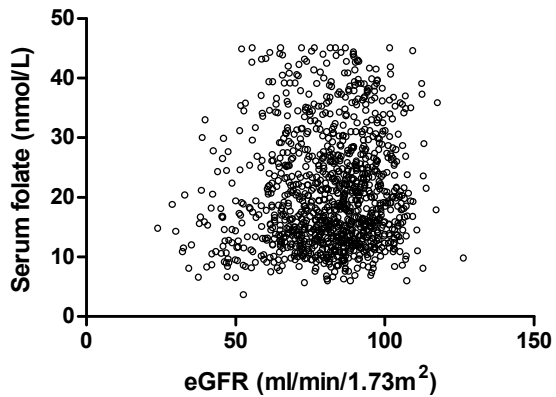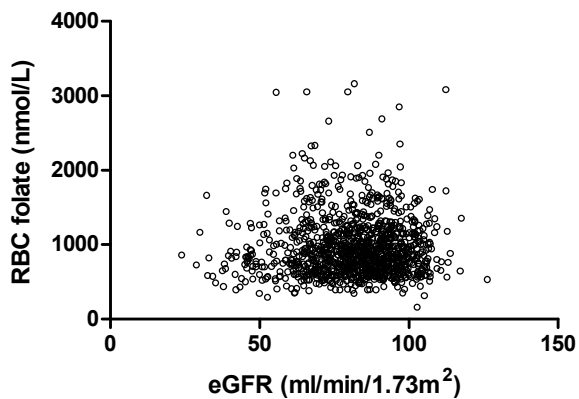

Supplement: Additional file 2: — Association of folate, vitamin B12 and related markers with kidney function (eGFR). The 6 panels show the serum concentrations of each of vitamin B12 (r=0.02;p=0.44), holoTC (r=−0.04; p=0.14) , MMA (r=−0.29; p<0.001), Hcy (r=−0.46; p<0.001), serum folate (r=0.10; p<0.001) and RBC folate (r=0.01; p=0.58) as compared to eGFR, plotted independently of age. [file 12877_2015_60_MOESM2_ESM.pdf]
